# Supplementary material for: Meta-Analysis and Gene Set Enrichment Relative to ER Status Reveal Elevated Activity of MYC and E2F in the “Basal” Breast Cancer Subgroup
Source: PLoS One. 2009 Mar 9;4(3):e4710. doi: 10.1371/journal.pone.0004710 (PMC2650420; doi:10.1371/journal.pone.0004710)
Supplement: Dataset S1 — GSEA results from first screen. The file contains a zipped archive of the HTML results of the second GSEA screen. Reports for enriched or depleted datasets may be accessed from the “index.html” link within the my_analysis.GseaPreranked.1219975950625.rpt.zip ZIP archive. Enrichment plots are available for each of the 50 most enriched and depleted gene sets. (13.32 MB ZIP) [file pone.0004710.s001.zip › my_analysis.GseaPreranked.1219975950625.rpt.zip!//V$E2F1_Q6_01.html]

Details for gene set V$E2F1\_Q6\_01[GSEA]

|  || Dataset | zSco.ERneg.to.ERpos\_collapsed |
| Phenotype | NoPhenotypeAvailable |
| Upregulated in class | na\_pos |
| GeneSet | V$E2F1\_Q6\_01 |
| Enrichment Score (ES) | 0.489571 |
| Normalized Enrichment Score (NES) | 2.1800544 |
| Nominal p-value | 0.0 |
| FDR q-value | 0.0 |
| FWER p-Value | 0.0 |
Table: GSEA Results Summary

  

Fig 1: Enrichment plot: V$E2F1\_Q6\_01      
 Profile of the Running ES Score & Positions of GeneSet Members on the Rank Ordered List

  

| PROBE | GENE SYMBOL | GENE\_TITLE | RANK IN GENE LIST | RANK METRIC SCORE | RUNNING ES | CORE ENRICHMENT || 1 | E2F3 |  |  | 6 | 7.369 | 0.0190 | Yes |
| 2 | NASP |  |  | 59 | 6.201 | 0.0313 | Yes |
| 3 | AK2 |  |  | 85 | 5.971 | 0.0452 | Yes |
| 4 | KLF5 |  |  | 87 | 5.966 | 0.0608 | Yes |
| 5 | GPRC5B |  |  | 103 | 5.845 | 0.0751 | Yes |
| 6 | CDC45L |  |  | 108 | 5.824 | 0.0901 | Yes |
| 7 | MCM4 |  |  | 180 | 5.436 | 0.0990 | Yes |
| 8 | MCM6 |  |  | 198 | 5.317 | 0.1118 | Yes |
| 9 | GMNN |  |  | 206 | 5.302 | 0.1252 | Yes |
| 10 | ORC1L |  |  | 212 | 5.280 | 0.1387 | Yes |
| 11 | PAX6 |  |  | 224 | 5.208 | 0.1516 | Yes |
| 12 | BMP2 |  |  | 275 | 5.006 | 0.1610 | Yes |
| 13 | CDC25A |  |  | 281 | 4.993 | 0.1738 | Yes |
| 14 | MCM7 |  |  | 289 | 4.970 | 0.1863 | Yes |
| 15 | TNF |  |  | 302 | 4.930 | 0.1984 | Yes |
| 16 | STMN1 |  |  | 310 | 4.907 | 0.2108 | Yes |
| 17 | EZH2 |  |  | 312 | 4.888 | 0.2236 | Yes |
| 18 | ARHGAP11A |  |  | 361 | 4.763 | 0.2325 | Yes |
| 19 | RANBP1 |  |  | 380 | 4.733 | 0.2436 | Yes |
| 20 | SYNCRIP |  |  | 402 | 4.679 | 0.2544 | Yes |
| 21 | SUV39H1 |  |  | 495 | 4.361 | 0.2589 | Yes |
| 22 | RAD51 |  |  | 508 | 4.332 | 0.2694 | Yes |
| 23 | PPRC1 |  |  | 513 | 4.325 | 0.2805 | Yes |
| 24 | TYRO3 |  |  | 516 | 4.316 | 0.2917 | Yes |
| 25 | MCM2 |  |  | 630 | 4.080 | 0.2938 | Yes |
| 26 | MCM3 |  |  | 656 | 4.019 | 0.3025 | Yes |
| 27 | AMD1 |  |  | 658 | 4.018 | 0.3130 | Yes |
| 28 | NCL |  |  | 678 | 3.977 | 0.3221 | Yes |
| 29 | RPS19 |  |  | 685 | 3.967 | 0.3321 | Yes |
| 30 | TMPO |  |  | 792 | 3.788 | 0.3340 | Yes |
| 31 | ILF3 |  |  | 801 | 3.774 | 0.3433 | Yes |
| 32 | MSH2 |  |  | 819 | 3.748 | 0.3519 | Yes |
| 33 | MYC |  |  | 1025 | 3.447 | 0.3454 | Yes |
| 34 | UNG |  |  | 1070 | 3.390 | 0.3510 | Yes |
| 35 | USP2 |  |  | 1158 | 3.290 | 0.3530 | Yes |
| 36 | FANCC |  |  | 1175 | 3.264 | 0.3604 | Yes |
| 37 | NOLC1 |  |  | 1222 | 3.217 | 0.3654 | Yes |
| 38 | PIK3CD |  |  | 1229 | 3.209 | 0.3734 | Yes |
| 39 | PRKDC |  |  | 1234 | 3.204 | 0.3815 | Yes |
| 40 | DNAJC9 |  |  | 1243 | 3.198 | 0.3894 | Yes |
| 41 | CDC5L |  |  | 1278 | 3.162 | 0.3951 | Yes |
| 42 | FBXO5 |  |  | 1304 | 3.137 | 0.4015 | Yes |
| 43 | NPAT |  |  | 1318 | 3.120 | 0.4087 | Yes |
| 44 | PCNA |  |  | 1346 | 3.087 | 0.4148 | Yes |
| 45 | HR |  |  | 1436 | 3.013 | 0.4160 | Yes |
| 46 | EFNA5 |  |  | 1488 | 2.953 | 0.4199 | Yes |
| 47 | ATAD2 |  |  | 1532 | 2.917 | 0.4243 | Yes |
| 48 | FLJ23861 |  |  | 1581 | 2.870 | 0.4282 | Yes |
| 49 | CASP2 |  |  | 1649 | 2.802 | 0.4305 | Yes |
| 50 | GNG4 |  |  | 1670 | 2.786 | 0.4363 | Yes |
| 51 | E2F1 |  |  | 1695 | 2.764 | 0.4418 | Yes |
| 52 | HIRA |  |  | 1696 | 2.763 | 0.4490 | Yes |
| 53 | PKMYT1 |  |  | 1715 | 2.753 | 0.4549 | Yes |
| 54 | FKBP5 |  |  | 1729 | 2.743 | 0.4612 | Yes |
| 55 | SUMO1 |  |  | 1736 | 2.736 | 0.4679 | Yes |
| 56 | CASP8AP2 |  |  | 1930 | 2.587 | 0.4601 | Yes |
| 57 | ATM |  |  | 1994 | 2.536 | 0.4619 | Yes |
| 58 | HMGA2 |  |  | 2058 | 2.478 | 0.4637 | Yes |
| 59 | RB1CC1 |  |  | 2258 | 2.345 | 0.4547 | Yes |
| 60 | FANCG |  |  | 2282 | 2.332 | 0.4591 | Yes |
| 61 | PPP1R8 |  |  | 2302 | 2.318 | 0.4638 | Yes |
| 62 | IPO7 |  |  | 2413 | 2.247 | 0.4614 | Yes |
| 63 | EIF1AX |  |  | 2473 | 2.215 | 0.4627 | Yes |
| 64 | RPL18 |  |  | 2487 | 2.204 | 0.4675 | Yes |
| 65 | SMARCA1 |  |  | 2501 | 2.199 | 0.4723 | Yes |
| 66 | HNRPD |  |  | 2585 | 2.156 | 0.4717 | Yes |
| 67 | FAF1 |  |  | 2600 | 2.144 | 0.4763 | Yes |
| 68 | CBX5 |  |  | 2621 | 2.133 | 0.4804 | Yes |
| 69 | PTMA |  |  | 2647 | 2.119 | 0.4841 | Yes |
| 70 | POLA2 |  |  | 2649 | 2.117 | 0.4896 | Yes |
| 71 | DNMT1 |  |  | 2844 | 2.017 | 0.4801 | No |
| 72 | NCBP1 |  |  | 2846 | 2.016 | 0.4854 | No |
| 73 | MAP4K1 |  |  | 3037 | 1.914 | 0.4760 | No |
| 74 | WEE1 |  |  | 3048 | 1.909 | 0.4802 | No |
| 75 | C10ORF119 |  |  | 3202 | 1.838 | 0.4735 | No |
| 76 | FXR2 |  |  | 3409 | 1.744 | 0.4624 | No |
| 77 | PAQR4 |  |  | 3434 | 1.736 | 0.4651 | No |
| 78 | TOPBP1 |  |  | 3475 | 1.722 | 0.4666 | No |
| 79 | DNAJC11 |  |  | 3676 | 1.628 | 0.4557 | No |
| 80 | MEIS2 |  |  | 3743 | 1.608 | 0.4549 | No |
| 81 | SLC9A7 |  |  | 3790 | 1.589 | 0.4556 | No |
| 82 | PRPS1 |  |  | 3889 | 1.556 | 0.4523 | No |
| 83 | POLE2 |  |  | 3975 | 1.520 | 0.4498 | No |
| 84 | STAG2 |  |  | 4224 | 1.421 | 0.4347 | No |
| 85 | UFD1L |  |  | 4248 | 1.414 | 0.4367 | No |
| 86 | RQCD1 |  |  | 4253 | 1.412 | 0.4401 | No |
| 87 | HNRPUL1 |  |  | 4310 | 1.387 | 0.4395 | No |
| 88 | CLSPN |  |  | 4483 | 1.324 | 0.4299 | No |
| 89 | RAB11B |  |  | 4771 | 1.230 | 0.4113 | No |
| 90 | STAG1 |  |  | 4794 | 1.225 | 0.4129 | No |
| 91 | ERF |  |  | 4869 | 1.199 | 0.4104 | No |
| 92 | HNRPR |  |  | 4913 | 1.185 | 0.4103 | No |
| 93 | DLEU1 |  |  | 4922 | 1.183 | 0.4128 | No |
| 94 | HTF9C |  |  | 4986 | 1.161 | 0.4111 | No |
| 95 | ARHGAP6 |  |  | 4991 | 1.159 | 0.4138 | No |
| 96 | MYH10 |  |  | 5076 | 1.124 | 0.4104 | No |
| 97 | FLI1 |  |  | 5103 | 1.114 | 0.4113 | No |
| 98 | EED |  |  | 5267 | 1.060 | 0.4017 | No |
| 99 | CHD4 |  |  | 5642 | 0.932 | 0.3758 | No |
| 100 | CCNT1 |  |  | 5647 | 0.931 | 0.3779 | No |
| 101 | ACO2 |  |  | 5713 | 0.907 | 0.3754 | No |
| 102 | ESPL1 |  |  | 5874 | 0.853 | 0.3654 | No |
| 103 | ADAMTS2 |  |  | 5988 | 0.817 | 0.3590 | No |
| 104 | UXT |  |  | 6065 | 0.791 | 0.3553 | No |
| 105 | ATP5G2 |  |  | 6071 | 0.791 | 0.3570 | No |
| 106 | YBX2 |  |  | 6299 | 0.716 | 0.3416 | No |
| 107 | C20ORF67 |  |  | 6482 | 0.657 | 0.3295 | No |
| 108 | WHSC1L1 |  |  | 6640 | 0.609 | 0.3192 | No |
| 109 | POLD3 |  |  | 6667 | 0.601 | 0.3188 | No |
| 110 | SFRS2 |  |  | 6677 | 0.597 | 0.3197 | No |
| 111 | TFAP4 |  |  | 6777 | 0.561 | 0.3137 | No |
| 112 | FZD1 |  |  | 6859 | 0.531 | 0.3089 | No |
| 113 | AP1S1 |  |  | 7316 | 0.377 | 0.2752 | No |
| 114 | GATA1 |  |  | 7431 | 0.334 | 0.2674 | No |
| 115 | MGC13053 |  |  | 7620 | 0.261 | 0.2538 | No |
| 116 | TBX6 |  |  | 7723 | 0.223 | 0.2467 | No |
| 117 | DLEU2 |  |  | 7765 | 0.204 | 0.2441 | No |
| 118 | SLC9A5 |  |  | 8303 | 0.000 | 0.2033 | No |
| 119 | MXD3 |  |  | 8312 | -0.002 | 0.2027 | No |
| 120 | ANKHD1 |  |  | 8331 | -0.007 | 0.2013 | No |
| 121 | HIST1H2BK |  |  | 8356 | -0.020 | 0.1995 | No |
| 122 | MTSS1 |  |  | 8381 | -0.029 | 0.1978 | No |
| 123 | MGAT2 |  |  | 8433 | -0.049 | 0.1940 | No |
| 124 | FLJ12529 |  |  | 8444 | -0.053 | 0.1934 | No |
| 125 | ACTN3 |  |  | 8512 | -0.084 | 0.1885 | No |
| 126 | SYNGR4 |  |  | 8638 | -0.147 | 0.1794 | No |
| 127 | GADD45B |  |  | 8774 | -0.199 | 0.1697 | No |
| 128 | SFRS1 |  |  | 8891 | -0.255 | 0.1615 | No |
| 129 | ID3 |  |  | 8993 | -0.308 | 0.1547 | No |
| 130 | PPM1D |  |  | 9078 | -0.350 | 0.1492 | No |
| 131 | CNOT3 |  |  | 9334 | -0.476 | 0.1311 | No |
| 132 | FLJ25476 |  |  | 9347 | -0.481 | 0.1314 | No |
| 133 | PCSK1 |  |  | 9351 | -0.483 | 0.1325 | No |
| 134 | MAZ |  |  | 9415 | -0.521 | 0.1291 | No |
| 135 | ET |  |  | 9587 | -0.611 | 0.1177 | No |
| 136 | ASXL2 |  |  | 9736 | -0.689 | 0.1082 | No |
| 137 | ZCWPW1 |  |  | 9773 | -0.709 | 0.1074 | No |
| 138 | GSPT1 |  |  | 9841 | -0.751 | 0.1043 | No |
| 139 | C9ORF97 |  |  | 9910 | -0.791 | 0.1012 | No |
| 140 | DDX17 |  |  | 9961 | -0.821 | 0.0995 | No |
| 141 | LRP1 |  |  | 10000 | -0.845 | 0.0989 | No |
| 142 | SPHK2 |  |  | 10057 | -0.878 | 0.0969 | No |
| 143 | MRPL40 |  |  | 10273 | -1.004 | 0.0832 | No |
| 144 | SP1 |  |  | 10401 | -1.092 | 0.0764 | No |
| 145 | NCOA6 |  |  | 10476 | -1.141 | 0.0738 | No |
| 146 | CTNND2 |  |  | 10581 | -1.227 | 0.0692 | No |
| 147 | HNRPA1 |  |  | 10609 | -1.250 | 0.0704 | No |
| 148 | SLC25A1 |  |  | 10628 | -1.267 | 0.0724 | No |
| 149 | POU2F1 |  |  | 10683 | -1.305 | 0.0717 | No |
| 150 | DDB2 |  |  | 10765 | -1.362 | 0.0691 | No |
| 151 | HMGN2 |  |  | 10916 | -1.492 | 0.0617 | No |
| 152 | C14ORF130 |  |  | 10945 | -1.523 | 0.0635 | No |
| 153 | INSM1 |  |  | 11073 | -1.642 | 0.0582 | No |
| 154 | AP4M1 |  |  | 11123 | -1.683 | 0.0589 | No |
| 155 | CTCF |  |  | 11128 | -1.687 | 0.0631 | No |
| 156 | PHF15 |  |  | 11215 | -1.755 | 0.0612 | No |
| 157 | HOXC10 |  |  | 11345 | -1.878 | 0.0563 | No |
| 158 | FHOD1 |  |  | 11408 | -1.941 | 0.0567 | No |
| 159 | TCF8 |  |  | 11586 | -2.111 | 0.0488 | No |
| 160 | USP52 |  |  | 11931 | -2.478 | 0.0292 | No |
| 161 | NUMA1 |  |  | 12034 | -2.614 | 0.0283 | No |
| 162 | TMEM15 |  |  | 12137 | -2.754 | 0.0278 | No |
| 163 | CXORF12 |  |  | 12144 | -2.762 | 0.0346 | No |
| 164 | SFRS12 |  |  | 12641 | -3.513 | 0.0062 | No |
| 165 | ERBB2IP |  |  | 12910 | -4.180 | -0.0032 | No |
| 166 | REPS2 |  |  | 13263 | -6.105 | -0.0139 | No |
| 167 | ENPP1 |  |  | 13307 | -6.873 | 0.0010 | No |
Table: GSEA details [plain text format]

  

Fig 2: V$E2F1\_Q6\_01: Random ES distribution      
 Gene set null distribution of ES for **V$E2F1\_Q6\_01**
